# Personenbezogene Gesundheitsdaten in einer Pandemie – ethische und rechtliche Aspekte

**DOI:** 10.1007/s10354-020-00785-8

**Published:** 2020-09-21

**Authors:** Martina Schmidhuber, Karl Stöger

**Affiliations:** 1 Professur für Health Care Ethics, Universität Graz, Heinrichstraße 78b/1, 8010 Graz, Österreich; 2 Professur für Öffentliches Recht, Universität Graz, Universitätsstraße 15/C3, 8010 Graz, Österreich

**Keywords:** Corona-Apps, COVID-19, Datenschutz, Ethik, Immunitätsnachweise, Recht, Risikozertifikate, Corona apps, COVID-19, Data protection, Ethics, Proof of immunity, Law, Risk certificates

## Abstract

Im Zusammenhang mit der COVID-19-Pandemie ist immer wieder das Argument zu hören gewesen, Gesundheitsschutz müsse dem Datenschutz jedenfalls vorgehen. In dieser Pauschalität ist diese Aussage zwar plakativ, aber weder aus ethischer noch aus rechtlicher Sicht zutreffend. Eine Pandemie kann zwar durchaus einen Grund dafür liefern, vorübergehend auf Gesundheitsdaten zuzugreifen. Dabei ist aber zum einen darauf zu achten, dass dies nur in unbedingt notwendigem Ausmaß erfolgt und zum anderen, in welchem größeren Kontext diese Daten verwendet werden. Dieser Beitrag skizziert an drei Beispielen – Risikoatteste, Tracking Apps und Immunitätszertifikate – einige der dabei zu berücksichtigenden ethischen und rechtlichen Überlegungen. Damit soll verdeutlicht werden, dass auch in der Pandemie die Abwägung von Argumenten und die Verhältnismäßigkeit der ergriffenen Maßnahmen nicht aus den Augen verloren werden dürfen.

## Summary

In the context of the COVID-19 pandemic, it has repeatedly been argued that the protection of (public) health should at any rate take precedence over data protection. This argument is bold and clear, but it is neither ethically nor legally correct. While the fight against a pandemic can indeed require a more comprehensive access to sensitive health data than usual, it must be ensured that such a measure is only taken to the extent absolutely necessary. Furthermore, the wider context in which the data will be used must also be taken into consideration. This article gives three examples—certificates for members of risk groups, tracking apps and immunity certificates—to discuss some of the ethical and legal considerations to be taken into account. The authors aspire to illustrate that even in a pandemic, the balancing of arguments and the need for a proportionality of measures taken must never be forgotten.

## Kein Datenschutz in der Pandemie?

Die COVID-19-Pandemie hat – nicht nur in Österreich – die Verwendung von persönlichen Gesundheitsdaten wieder zu einem intensiver diskutierten Thema gemacht. Der Zugriff sowohl staatlicher als auch privater Einrichtungen auf personenbezogene Gesundheitsdaten wird dabei in der Öffentlichkeit durchaus kritisch bewertet, wie etwa die Diskussion um die verpflichtende Verwendung der „Corona-App“ des Roten Kreuzes zeigt. Diese Diskussion ist umso bemerkenswerter, da Menschen außerhalb der Situation einer Pandemie mitunter sehr unbekümmert mit persönlichen Gesundheitsdaten umgehen. So stellen etwa im Freizeitbereich Nutzer von Gesundheits-Apps diesen zahlreiche, durchaus aussagekräftige Daten leichtfertig zur Verfügung. Insoweit könnte die COVID-19-Pandemie zumindest kurzfristig zur verstärkten Wachsamkeit hinsichtlich der Verwendung von Gesundheitsdaten beigetragen haben, wie sie seit Jahren in der Literatur propagiert wird [1]. Im Zusammenhang mit COVID-19 wurde nicht nur im politischen Diskurs das Argument gebracht, Datenschutz müsse in einer Pandemie jedenfalls hinter den Gesundheitsschutz zurücktreten (z. B. [2] mit Zitat von Bundeskanzler Kurz). In dieser Pauschalität ist diese Aussage zwar plakativ, aber weder aus ethischer noch aus rechtlicher Sicht [3] zutreffend. Zugleich ist es so, dass in den letzten Wochen in Österreich durchaus neue rechtliche Grundlagen für Datenübermittlungen zur Eindämmung der Pandemie geschaffen und andere diskutiert wurden. Der vorliegende Beitrag möchte den groben ethischen und rechtlichen Rahmen solcher Maßnahmen skizzieren und dabei vor allem vier Dinge deutlich machen. Erstens: Sowohl aus ethischer als auch aus rechtlicher Sicht ist eine Pandemie ein durchaus tauglicher Grund, nicht nur dem Staat, sondern auch Privaten den Zugang zu Gesundheitsdaten zu eröffnen. Zweitens: Dabei darf aber nicht übersehen werden, dass dieser Zugang auf das erforderliche Mindestmaß beschränkt sein muss, was insbesondere bedeutet, dass Eigenverantwortlichkeit auch in der Pandemie ein zentraler ethischer und rechtlicher Wert ist. Drittens: Das eigentliche Problem liegt oft gar nicht im Zugriff auf die Gesundheitsdaten als solches, sondern darin, dass sich die Verarbeitung dieser Daten im Ergebnis auch zu Lasten Dritter auswirken kann. Viertens plädieren wir im Fazit dafür, dass mit dem Verschwinden der Pandemie solchen Datenverarbeitungen auch wieder ein Ende gesetzt werden muss.

Diese Überlegungen sollen anhand von drei Beispielen verdeutlicht werden, die in Österreich bereits teilweise verwirklicht, teilweise (zumindest indirekt) geplant oder in Diskussion sind: Risikozertifikate zur Freistellung, Infektionsmeldungen mittels einer Corona-App und Immunitätszertifikate als Nachweis einer durchgemachten Erkrankung. In allen drei Fällen werden gesundheitsbezogene und damit besonders schutzwürdige Daten verarbeitet: Das Risikozertifikat bestätigt das Vorliegen einer bestimmten, wenn auch nicht näher bestimmten Vorerkrankung, die Corona-App verarbeitet unter anderem die Meldung einer Person über das Vorliegen einer Infektion (und im Übrigen zahlreiche Kontakte mit anderen Personen) und das Immunitätszertifikat bestätigt die überstandene COVID-19-Erkrankung. Besonders zu beachten ist, dass, wie bereits erwähnt, die Verarbeitung dieser Daten auch noch Auswirkungen auf andere Personen hat: Das Risikozertifikat verpflichtet den Arbeitgeber insbesondere, Mitarbeitern Home-Office zu ermöglichen oder besondere Maßnahmen zu ihrem Schutz (bis hin zur Freistellung) zu treffen. Wenn jemand die Mitteilung erhält, dass ein Corona-App-Kontakt infiziert ist, wird sich diese Person in freiwillige Quarantäne begeben müssen, da man ihr sonst allenfalls eine fahrlässige Verbreitung einer ansteckenden Krankheit – immerhin eine gerichtlich strafbare Handlung – vorwerfen könnte. Und bei den Immunitätszertifikaten gibt es jedenfalls außerhalb Österreichs Überlegungen, deren Inhabern eventuell besondere Rechte zuzugestehen – eine ethisch und verfassungsrechtlich durchaus problematische Besserstellung, wenn man bedenkt, dass eine „durchgemachte“ Erkrankung nicht mit einer „auf Anforderung“ erhaltenen Impfung vergleichbar ist.

## Ethische und rechtliche Eckpunkte

Aus ethischer Sicht ist für die Verwendung von personenbezogenen Daten für Zwecke der Bekämpfung einer Pandemie eine Abwägung zwischen Autonomie (im Sinne der Herrschaft über die eigenen Daten) und Gemeinwohl erforderlich. Individuen haben grundsätzlich die Hoheit über ihre Gesundheitsdaten, wie auch das Beispiel der elektronischen Gesundheitsakte (ELGA) in Österreich zeigt, von der man sich abmelden kann (sogenannte Opt-out-Möglichkeit) [4]. Die Pandemie als Ausnahmezustand führte allerdings seit Mitte März 2020 vor Augen, dass es nicht nur um die Autonomie und die Gesundheit der einzelnen Person geht, sondern um die Gesundheit vieler Menschen (Public Health). Eine ansteckende Krankheit geht aus ethischer Sicht nicht nur den Einzelnen an, wie auch an anhaltenden Debatten zur Masern-Impfung immer wieder deutlich wird [5, 6]. Ein Staat kann deshalb gute Gründe haben, die Autonomie des Einzelnen zugunsten des Gemeinwohls einzuschränken. Aus ethischer Sicht stellt sich jedoch die Frage, nach welchen Maßstäben hier zu handeln ist. Die klassischen Formen der deontologischen Ethik und des Konsequentialismus bzw. Utilitarismus scheinen verkürzt und unbefriedigend: In der deontologischen Ethik, der „Pflichtenethik“, wird die moralische Güte einer Handlung daran gemessen, ob sie erlaubt oder verboten ist. So fachte der prominenteste Vertreter der deontologischen Ethik, Immanuel Kant, mit seinem Lügenverbot viele Diskussionen an, weil er dieses in jeder Situation hochhielt. Problematisch an der deontologischen Ethik ist, dass die Rahmenbedingungen zu wenig Berücksichtigung finden, weil nur die Pflicht etwas zu tun oder nicht zu tun zählt. Wenn nun im Kontext der Covid-19-Krise allein die Pflicht leben zu retten in den Mittelpunkt der Überlegungen gestellt wird, kann dies zu anderen Problemen führen, wie etwa zu enormen wirtschaftlichem Schaden oder zu Fällen, in denen Patienten, die nicht an Covid-19 erkrankt sind, aufgrund der Priorisierung nicht behandelt werden und dadurch andere körperliche Schäden erleiden.

Im Konsequentialismus, dessen bekannteste Form der Utilitarismus ist, kommt es auf das Ergebnis einer Handlung an. Eine Handlung ist dementsprechend dann moralisch gut, wenn sie Gutes maximiert und Schlechtes minimiert. In anderen Worten: Es geht um das größte Glück möglichst vieler Menschen, auch wenn dies zu Lasten einiger weniger geschieht. Im Falle der Covid-19-Krise hieße das, dass man das Leben einer größtmöglichen Anzahl von Menschen schützen bzw. retten und die Kosten des Gesundheitssystems minimieren muss.

Dieser Einblick in die zwei wichtigsten ethischen Theorien soll gezeigt haben, dass beide auf ihre Art nicht zufriedenstellend sind: Lediglich die Pflicht, etwas zu tun, vernachlässigt die Rahmenbedingungen (deontologische Ethik) und die alleinige Berücksichtigung der Konsequenz einer Handlung greift zu kurz, weil der Weg zum Ergebnis vernachlässigt wird (Utilitarismus). Nicht zuletzt aufgrund dieser Mängel hat sich in medizinethischen Fragestellungen und in Public Health Ethics die Prinzipienethik nach Beauchamp und Childress [7] etabliert. Die vier gleichwertigen Prinzipien Autonomie, Fürsorge, Nicht-Schaden und Gerechtigkeit müssen in Konfliktsituation stets ausbalanciert werden. Die Anwendung der Prinzipienethik in der Covid-19-Krise ist in erster Linie mit der Frage verbunden, welche Autonomieeinschränkungen zugunsten des Gemeinwohls gerecht sind. Aus ethischer Sicht sind die drei folgenden Beispiele genau danach zu beurteilen.

Ethische und rechtliche Überlegungen decken sich bekanntlich nicht immer, gerade im Falle der Datenverarbeitung zur Bekämpfung einer grenzüberschreitenden Gesundheitsgefährdung allerdings besteht hier durchaus Gleichklang. So betrifft die Verwendung von Gesundheitsdaten Grundrechte, die sowohl auf nationaler Ebene als auch auf europäischer Ebene verankert sind, nämlich das Recht auf Schutz des Privatlebens (Artikel 8 der Europäischen Menschenrechtskonvention [EMRK], Artikel 7 der EU-Grundrechtecharta [EU-GRC]) und das Grundrecht auf Datenschutz (§ 1 des Datenschutzgesetzes, Artikel 8 der EU-GRC). Bei beiden Grundrechten ist allerdings anerkannt, dass sie aus Gründen des Gesundheitsschutzes eingeschränkt werden können. So erwähnt etwa Artikel 8 der EMRK das Schutzgut der „öffentlichen Gesundheit“ als Grund für eine Einschränkung dieses Grundrechts. Fast noch wichtiger ist allerdings, dass die Datenschutz-Grundverordnung [DSGVO] als europaweit weitgehend einheitlicher Rahmen für Datenverwendungen gerade grenzüberschreitende Gesundheitsgefahren wie Pandemien ausdrücklich als einen Grund angibt, der die Verarbeitung sensibler Daten wie Gesundheitsdaten rechtfertigen kann. Erwägungsgrund 46 der DSGVO erklärt dazu sehr deutlich: „Die Verarbeitung personenbezogener Daten sollte ebenfalls als rechtmäßig angesehen werden, wenn sie erforderlich ist, um ein lebenswichtiges Interesse der betroffenen Person oder einer anderen natürlichen Person zu schützen. Personenbezogene Daten sollten grundsätzlich nur dann aufgrund eines lebenswichtigen Interesses einer anderen natürlichen Person verarbeitet werden, wenn die Verarbeitung offensichtlich nicht auf eine andere Rechtsgrundlage gestützt werden kann. Einige Arten der Verarbeitung können sowohl wichtigen Gründen des öffentlichen Interesses als auch lebenswichtigen Interessen der betroffenen Person dienen; so kann beispielsweise die Verarbeitung für humanitäre Zwecke einschließlich der Überwachung von Epidemien und deren Ausbreitung oder in humanitären Notfällen insbesondere bei Naturkatastrophen oder vom Menschen verursachten Katastrophen erforderlich sein“. Damit wird die Situation beschrieben, die gerade für COVID-19 prägend ist: Zwar stellt die Krankheit auch für den Einzelnen, insbesondere für Angehörige von Risikogruppen, eine gesundheitliche Bedrohung dar, ihre größte Gefahr liegt jedoch darin, dass zu viele, gleichzeitig behandlungsbedürftige Personen das Gesundheitssystem „überlasten“. Dies ist dann nicht nur für an COVID-19 erkrankte Personen nachteilig, sondern auch für solche, die eine andere medizinische Behandlung benötigen. Eigenschutz ist hier immer auch Fremdschutz.

Artikel 9 DSGVO, der die Verarbeitung (unter anderem) von Gesundheitsdaten erlaubt, sieht dementsprechend neben dem (ethisch besonders wünschenswerten) Fall der Zustimmung des Betroffenen auch noch diverse andere Fälle vor, in denen es auch gegen den Willen einer betroffenen Person zur Verarbeitung von Gesundheitsdaten kommen kann: Möglich ist das etwa, damit der Arbeitgeber gesetzliche Verpflichtungen gegenüber einem Arbeitnehmer erfüllen kann, wenn es um den Schutz lebenswichtiger Interessen geht und eine Person (etwa wegen einer Erkrankung) nicht zustimmen kann, zu Zwecken der Gesundheitsvorsorge oder medizinischen Behandlung und schließlich aus „Gründen des öffentlichen Interesses im Bereich der öffentlichen Gesundheit, wie dem Schutz vor schwerwiegenden grenzüberschreitenden Gesundheitsgefahren“. All dies scheint auf den ersten Blick zu bestätigen, dass der Datenschutz in der Pandemie von untergeordneter Bedeutung ist. Wie bereits oben erwähnt, greift dieser Schluss aber zu kurz. Eingriffe in Grundrechte – nicht nur das auf Datenschutz – müssen verhältnismäßig sein, und auch die DSGVO erwähnt ausdrücklich das Gebot, dass die Verarbeitung von Daten auf „das für die Zwecke der Verarbeitung notwendige Maß beschränkt“ bleiben muss („Datenminimierung“ nach Artikel 5 Absatz 1 litera c DSGVO). Diese rechtliche Verhältnismäßigkeitsprüfung entspricht im vorliegenden Fall weitestgehend der ethischen Abwägung zwischen der Autonomie des Einzelnen und dem Gemeinwohl.

Zusammenfassend ist daher festzuhalten, dass sowohl aus ethischer als auch rechtlicher Sicht der Umgang mit Daten in einer Pandemie eine genaue Abwägung von (gegenläufigen) Prinzipien und Interessen erfordert. Dabei ist grundsätzlich jeder Fall der Datenverwendung für sich zu beurteilen, von einem absoluten Vorrang des Gesundheitsschutzes vor dem Datenschutz kann daher keine Rede sein. Gemeinsam ist aber allen Fällen, dass auch in der Pandemiebekämpfung das Gebot „Nicht mehr als nötig“, welches etwa auch für die Dauer der Aufbewahrung der Daten ausschlaggebend ist, letztlich den entscheidenden Maßstab bildet. Hier geht es um eine ethische bzw. rechtliche Abwägung, in der auch unterschiedliche nationale Verständnisse dessen sichtbar werden, wie der Wert der Privatheit beurteilt wird bzw. wie weit Grundrechte reichen. Darauf wird im Zusammenhang mit der Corona-App noch zurückzukommen sein. Zugleich ist gerade die Verhältnismäßigkeit einer Verarbeitung von Gesundheitsdaten in der ersten Phase des Lockdowns fast zu wenig betont worden, inzwischen ist das Bewusstsein dafür aber wieder gestiegen und hat sich auch in den einschlägigen (ethischen und rechtlichen) Diskussionen niedergeschlagen.

## Achtsamer Umgang mit Daten auch bei ansteckenden Krankheiten: Drei Beispiele

Die besondere Bedeutung der Beschränkung auf das notwendige Ausmaß und mögliche negative Folgen ihrer Missachtung sollen nunmehr an den bereits erwähnten drei Beispielen gezeigt werden:

Das COVID-19-Risikoattest ist ethisch und rechtlich brisanter, als es auf den ersten Blick wirkt. Mit den Worten zweier einschlägig ausgewiesener Juristen: Dem Arbeitgeber Einblick in den Gesundheitszustand seiner Arbeitnehmer zu gewähren, war „bisher arbeitsrechtlich und datenschutzrechtlich verpönt“ [8]. Nunmehr aber erhält ein Arbeitnehmer auf Wunsch eine ärztliche Bestätigung, die über seine Zugehörigkeit zu einer Risikogruppe (z. B. aufgrund von Diabetes, Asthma, etc.) Aufschluss gibt und die er dem Arbeitgeber vorlegen kann, was diesen dann zur Ergreifung von Schutzmaßnahmen (insbesondere Ermöglichung von Home-Office; allenfalls Freistellung) verpflichtet. Nun legt der Arbeitnehmer zwar die Bestätigung von sich aus vor, dennoch besteht keine uneingeschränkte Freiwilligkeit, da der Arbeitnehmer zur Vermeidung einer Gesundheitsgefährdung unter Zugzwang geraten wird, den besonderen Schutz zu begehren [8]. Dennoch – und das ist ausdrücklich zu begrüßen – wird mit den Daten im Lichte des gesetzten Ziels – Schutz des Arbeitnehmers und des Gesundheitssystems – sehr zurückhaltend vorgegangen. In einem ersten Schritt wird der Angehörige der Risikogruppe auf Grundlage von vorhandenen Medikationsdaten direkt durch die Sozialversicherung verständigt, das heißt hier ist noch kein Dritter involviert. Da die Treffgenauigkeit der Medikationsdaten durchaus fraglich sein kann [4], obliegt die endgültige Entscheidung über die Zugehörigkeit zur Risikogruppe einem der beruflichen Verschwiegenheit unterliegenden Angehörigen des ärztlichen Berufs. Erst das von diesem ausgestellte Attest gelangt dann durch Vorlage des Arbeitnehmers an den Arbeitgeber als Dritten, wobei dieser dann zu einem besonders sorgfältigen Umgang mit diesen Daten verpflichtet ist und Angehörige einer Risikogruppe wegen ihres Gesundheitszustandes keinesfalls kündigen darf [8, 9]. Damit soll auch die Angst vor der Preisgabe dieser heiklen Information genommen werden. Auch wenn durchaus noch Fragen offen bleiben [8, 9], gelingt der Regelung die grundsätzliche Balance zwischen Datenschutz und Schutz sowohl des Einzelnen als auch des Gesundheitssystems und dadurch auch des Gemeinwohls: So erfolgt der Schutz der Risikogruppe nicht nur in deren eigenem, sondern im allgemeinen Interesse, zugleich haben es aber die Angehörigen der Risikogruppe selbst in der Hand, wie weit sie diesen Schutz in Anspruch nehmen. Dies entspricht den rechtlichen Vorgaben an eine ausgewogene Interessenabwägung. Ein ähnliches Ergebnis zeigt sich aus ethischer Sicht: Im Sinne der Prinzipienethik wird in diesem Fall sowohl der Autonomie des Einzelnen, indem er auf Wunsch geschützt wird, Rechnung getragen, als auch der Gerechtigkeit, indem das Gesundheitssystem durch Präventivmaßnahmen (Home-Office, Freistellung) entlastet wird.

Deutlich heikler – und schon deswegen mehr diskutiert, weil es ein international verbreitetes Thema ist – sind Corona-Apps, die nicht ohne Grund als „Stresstest für Privatheit und Datenschutz“ [10] bezeichnet wurden. Bei ihnen ist die Übermittlung eines gesundheitsbezogenen Datums – nämlich der vermuteten bzw. bestätigten COVID-19-Infektion – nur ein kleiner Teil der Funktionsweise, der jedoch in Kombination mit den Kontaktdaten der zu informierenden Personen erhebliche Wirkungen auslöst, nämlich eine Verpflichtung der informierten Personen, sich vorläufig als „potenziell infiziert“ zu betrachten. Daraus folgt auch die Notwendigkeit der Selbstisolierung, wollen sich diese Personen bei tatsächlicher Infektion nicht etwa strafrechtlichen Folgen (fahrlässige Gefährdung von Personen durch übertragbare Krankheiten; § 179 Strafgesetzbuch) aussetzen [11]. Zugleich bewirkt diese Datenübermittlung auch aus Sicht des Infizierten eine potenzielle Pflicht zur Offenlegung seiner Erkrankung, wird doch für manche Kontakte durchaus ermittelbar sein, wer die infizierte Person ist. Mit anderen Worten: Die Corona-Apps sind gerade deswegen ein so kontroversieller Diskussionspunkt, weil von ihnen sehr viele Personen betroffen sein können (die infizierte Person und deren Kontakte). Als besonders problematisch würde sich daher ihre verpflichtende Verwendung erweisen: Auf Grund der erheblichen Folgen einer nicht einwandfreien Funktion der App, die möglicherweise einen Fehlalarm auslösen könnte und dann eine Person, angesichts möglicher strafrechtlicher Folgen, letztlich zur Selbstisolierung „zwingt“, sind falsch-positive Meldungen bei Corona-Apps ein nicht zu unterschätzender Faktor [12]. Darüber hinaus gilt es zu bedenken, dass ein Teil der gefährdeten Gruppe, nämlich jene mit einem Lebensalter über 65 Jahre, möglicherweise kein Smartphone besitzt. Eine deutsche Studie aus dem Jahr 2016 zeigt, dass zwar 75 % der Menschen, die bereits 70 Jahre und älter sind, ein Mobiltelefon nutzen, aber lediglich 14 % davon ein Smartphone [13]. Werden etwa bestimmte Begünstigungen an die zwingende Verwendung einer Corona-App geknüpft, ist dies ethisch und rechtlich in Hinblick auf eine Altersdiskriminierung besonders heikel. Das bedeutet allerdings nicht, dass eine solche verpflichtende Verwendung völlig ausgeschlossen ist, einige demokratisch organisierte Staaten außerhalb Europas haben sie auch verwirklicht (z. B. Südkorea, Israel oder Singapur [10, 14]). Hier kommt ein durchaus unterschiedliches Verständnis der Gewichtung bestimmter Grundrechte, wie etwa dem Datenschutz und dem Lebensschutz, in unterschiedlichen Teilen der Welt zum Ausdruck. Innerhalb der EU wird bislang auf die freiwillige Verwendung solcher Apps gesetzt, und das mit gutem Grund: Eine solche freiwillige Verwendung von Corona-Apps, bei der die Benutzer sich auf Grund entsprechender Informationen bewusst sind, worauf sie sich einlassen, ist rechtlich gesehen auf Grund der oben erwähnten Offenheit der DSGVO für die Interessen des Gesundheitsschutzes in Europa teilweise leichter verwirklichbar als in anderen Rechtsordnungen, die in der allgemeinen Wahrnehmung als weniger streng in Datenschutzfragen gelten (zum Vergleich zwischen der EU und den USA bzw. zusätzlich Kalifornien, vgl. [10]). Dies kommt auch in einer Stellungnahme des Europäischen Datenschutzausschusses zum Ausdruck, in dem dieser der freiwilligen Verwendung solcher Apps unter Einhaltung entsprechender Sicherheitsvorkehrungen zum Schutz der Daten aller Betroffenen grundsätzlich nicht ablehnend gegenübersteht [15]. Dennoch: Sowohl ethisch als auch rechtlich sind Corona-Apps ein anschaulicher Beweis dafür, dass Maßnahmen zur Sicherung der öffentlichen Gesundheit keinesfalls zu einer völligen Beseitigung des Datenschutzes führen dürfen. Dass sich Gesundheits-Apps immer größerer Beliebtheit erfreuen – etwa auch zur Sekundärprävention nach einem Schlaganfall [16] – legt den Schluss nahe, dass es sich bei diesen Apps um Instrumente handelt, die Menschen nicht nur für ihre eigene Gesundheit, sondern auch zugunsten des Gemeinwohls annehmen. Umso wesentlicher ist es, dass sie in doppelter Hinsicht sicher sind: Einerseits muss gewährleistet werden, dass sie technisch sicher funktionieren, um die oben erwähnten Fehlermeldungen zu vermeiden und andererseits aus datenschutzrechtlicher Sicht so sicher sind, wie dies vermittelt wird [17]. Wenn diese Voraussetzungen aber gegeben sind, dann ist die Verwendung dieser Apps auch verhältnismäßig im Sinne des Gebotes, dass Daten nur im unbedingt erforderlichen Ausmaß zur Erreichung bestimmter Ziele verwendet werden sollen. Dabei ist es im Lichte der Autonomie des Einzelnen besonders wichtig, dass die App freiwillig bleibt. Wenn sie freiwillig genutzt wird, etwa weil die Nutzer von der Sicherheit und Bedeutung überzeugt sind, wird zur Gerechtigkeit im Gesundheitswesen beigetragen, weil auf diese Weise Krankheitsfälle reduziert werden können und das Gesundheitssystem weniger belastet wird.

Zuletzt soll als dritte Maßnahme einer Verwendung von Gesundheitsdaten auf die in Österreich noch wenig diskutierten Immunitätszertifikate eingegangen werden. Besonders in Deutschland, aber auch in anderen Staaten ist die Diskussion um diese, aber auch deren kritische Bewertung, schon ein Stück weiter [18]. Wenn allerdings in Österreich flächendeckende, regelmäßige Testungen im Tourismussektor angekündigt werden, ist es bis zu diesen Zertifikaten nur ein kleiner Schritt: Warum soll eine Person regelmäßig getestet werden, wenn von ihrer Immunität auszugehen ist? Dies wird primär von der – hier nicht zu behandelnden – medizinischen Frage abhängen, ob eine durchgemachte Erkrankung wirklich ausschließt, andere zu infizieren. Auch Immunitätszertifikate betreffen gesundheitsbezogene persönliche Daten, da sie eine durchgemachte Erkrankung bekannt machen. Ethisch und rechtlich heikel sind sie aber vielmehr auf Grund der an sie geknüpften Folgen: Wird etwa eine durchgemachte Infektion mit besonderen Privilegien verknüpft, ist das ethisch regelmäßig nicht fair und rechtlich im Lichte des nicht nur in der österreichischen Verfassung enthaltenen Gleichheitsgrundsatzes sehr schnell diskriminierend. Aus ethischer Sicht darf der einzige Grund für Immunitätszertifikate nur der Schutz von Menschen sein. Weder Privilegien noch Diskriminierungen dürfen Folgen dieser Zertifikate sein. Vor allem bei Personen in Berufen mit hohem Kundenkontakt erscheint es im Sinne des Gemeinwohls sinnvoll, dass diese gesund und im besten Fall nachweislich immun sind. Dadurch sind sie selbst nicht mehr in Gefahr, angesteckt und sogenannte „Super-Spreader“ zu werden, wodurch wiederum vermieden werden kann, dass viele andere Menschen angesteckt werden. Für Personen, die nicht immun und in einem Beruf mit hohem Kundenkontakt tätig sind, gelten die vorgegebenen Abstandsregeln und der Mund-Nasen-Schutz sowie das eigenverantwortliche Handeln bei Krankheitssymptomen zu Hause zu bleiben. Dass man sich eine wiederholte Testung erspart, ist sachlich begründbar, aber etwa den Besuch von Veranstaltungen oder die Einreise mit einer bestehenden Immunität zu verknüpfen (und nicht nur mit der Abwesenheit einer Erkrankung), ist eine Diskriminierung. Im Gegensatz zu einer Impfung, die man bewusst in Anspruch nehmen kann, ist eine Erkrankung letztlich ein schicksalhaftes Ereignis. An sie Privilegien zu knüpfen, könnte letztlich einen perversen – und sowohl für den Einzelnen als auch für das Gesundheitssystem problematischen – Anreiz zur Selbstinfektion liefern [18]. Nicht weniger problematisch wäre es aber, aus der Immunität faktisch oder rechtlich besondere Verpflichtungen abzuleiten: Eben weil eine Erkrankung schicksalhaft ist, wäre es bedenklich, „immunes“ Gesundheitspersonal mit geringerem Schutz auszustatten oder überhaupt primär zur Versorgung von COVID-19-Erkrankten einzuteilen. Derartige Maßnahmen werden selbst in einer Pandemie nur in Ausnahmefällen rechtfertigbar sein, etwa um einen völligen Zusammenbruch des Systems zu verhindern. Abgesehen davon darf das Wissen um eine bestehende Immunität zwar berücksichtigt, nicht aber für sich zu einem Kriterium bei der Verteilung von dienstlichen Aufgaben werden.

Darüber hinaus sollte es freilich selbstverständlich sein, dass auch die Immunzertifikate datenschutzrechtlich sensibel behandelt werden und diese nach der Pandemie keine Rolle mehr spielen. Ein Slippery-Slope-Effekt wäre zu diagnostizieren, wenn (über die Immunitätszertifikate hinausgehend) Gesundheitszertifikate als grundsätzlich sinnvoll erachtet werden und sich etwa in Richtung einer Kontrolle über den psychischen Zustand von Arbeitnehmern ausbreiten würde, z. B. der Arbeitgeber Bescheid weiß, ob jemand depressive Symptome hat und deshalb weniger leistungsfähig sein könnte.

Auch im Hinblick auf eine mögliche, von vielen bald erhoffte Impfung gegen COVID-19 stellt sich die Frage, ob Immunzertifikate dann eine Rolle spielen würden. Wären jene, die als immun gelten, die letzten die ein Recht auf eine Impfung hätten? Was aber, wenn das Ergebnis eines Immunitätstests falsch-positiv ist [19]? Zudem scheint noch nicht klar zu sein, wie lange die Immunität tatsächlich anhält. Deshalb ist es erforderlich, auch hier einer Diskriminierung von Menschen, die als immun gelten, ganz bewusst entgegenzuwirken.

Immunitätszertifikate erweisen sich damit sowohl aus ethischer als auch aus rechtlicher Sicht als die heikelste der drei hier vorgestellten Maßnahmen unter Verwendung von Gesundheitsdaten. Rechtlich, aber auch ethisch lässt sich auf Grund der Prinzipien der Gerechtigkeit bzw. des Gemeinwohls zwar überzeugend argumentieren, dass einzelne Personen Maßnahmen, die ihnen nicht schaden (ein simpler Antikörpertest) nicht verweigern sollten, wenn dadurch (etwa weil die zu testenden Personen in intensivem Kundenkontakt arbeiten) die Ansteckung anderer Menschen erfolgreich verhindert werden könnte. Hier würden der Datenschutz bzw die Autonomie des Einzelnen im Interesse der öffentlichen Gesundheit bzw. der Gerechtigkeit an ihre Grenzen stoßen. Dazu müsste aber klar sein, welches Ziel mit der Verwendung von Immunitätszertifikaten aus Sicht des Gesundheitsschutzes überhaupt erreicht werden soll und ob dazu nicht brauchbarere Alternativen bestehen. Diese Fragen sind aber bisher (auch im politischen Diskurs) noch nicht ausreichend erörtert worden. Schließlich dürfte der Einsatz von Immunitätszertifikaten, wie bereits erwähnt, keinesfalls zu spürbaren Nachteilen der als immun erkannten Personen (z. B. geringer Schutz vor Exposition) führen. Anders gesprochen: Nur wenn die Verwendung von Immunitätszertifikaten überzeugenden gesundheitlichen Nutzen für die Allgemeinheit bringt und nicht zu Diskriminierungen oder Privilegierungen führt, lässt sich die damit verbundene Verwendung von Gesundheitsdaten rechtlich und ethisch rechtfertigen. Gerade die Vielschichtigkeit der dargestellten Probleme im Zusammenhang mit der Verwendung von Immunitätszertifikaten lässt dies aber zum derzeitigen Zeitpunkt durchaus fraglich erscheinen.

## Fazit: Nicht mehr als nötig und nur solange wie nötig

Die vorangegangen Überlegungen sollten verdeutlichen, dass Gesundheitsdaten auch in der Pandemie mit Sorgfalt zu behandeln sind, wenn man ethische und rechtliche Probleme vermeiden will. Diese können nicht nur die Person treffen, von der die Daten stammen, sondern auch Dritte können auf Grund einer Datenverarbeitung mit erheblichen Folgen konfrontiert sein (z. B. Pflicht zur Selbstisolation). Auch wenn in einer Pandemie die Verarbeitung von mehr Gesundheitsdaten rechtfertigbar ist als außerhalb einer solchen, sind die Folgen der Verarbeitung – und damit die Verhältnismäßigkeit der Datenverarbeitung in Hinblick auf das angestrebte Ziel – stets im Auge zu behalten. Und dies führt zu einem letzten, sowohl in ethischer als auch in rechtlicher Hinsicht zu betonenden Punkt: Sobald die COVID-19-Pandemie überstanden ist, gibt es auch keine besonderen Gründe mehr, die Risikoatteste, Tracking-Apps und Immunitätszertifikate rechtfertigen können (vgl. [20]). Es ist sehr zu hoffen, dass man sich nicht dauerhaft an diese gewöhnen muss, sondern dass in absehbarer Zeit eine medizinische Antwort auf die Herausforderungen von COVID-19 gefunden wird und Menschen bis dahin eigenverantwortlich im Sinne ihrer eigenen Gesundheit und der ihrer Mitmenschen handeln.
